# Zirconia implants and peek restorations for the replacement of upper molars

**DOI:** 10.1186/s40729-016-0062-2

**Published:** 2017-02-20

**Authors:** José María Parmigiani-Izquierdo, María Eugenia Cabaña-Muñoz, José Joaquín Merino, Arturo Sánchez-Pérez

**Affiliations:** 1 Periodontics Unit, Faculty of Medicine and Dentistry, University of Murcia (Spain), Murcia, Spain; 2 Clínica CIROM, Murcia, 30001 Spain; 3 Clínica Odontologíca Universitaria, Hospital Morales Meseguer, 2ª planta, C/ Marqués de los Vélez s/n, Murcia, 30008 Spain

**Keywords:** Dental implants, Zirconia, Osseointegration, Ceramics, Polyether ketone, Elastic modudus

## Abstract

**Background:**

One of the disadvantages of the zirconia implants is the lack of elasticity, which is increased with the use of ceramic or zirconia crowns. The consequences that could result from this lack of elasticity have led to the search for new materials with improved mechanical properties.

**Case presentation:**

A patient who is a 45-year-old woman, non-smoker and has no medical record of interest with a longitudinal fracture in the palatal root of molar tooth 1.7 and absence of tooth 1.6 was selected in order to receive a zirconia implant with a PEEK-based restoration and a composite coating. The following case report describes and analyses treatment with zirconia implants in molars following a flapless surgical technique. Zirconia implants are an alternative to titanium implants in patients with allergies or who are sensitive to metal alloys. However, one of the disadvantages that they have is their lack of elasticity, which increases with the use of ceramic or zirconia crowns. The consequences that can arise from this lack of elasticity have led to the search for new materials with better mechanical properties to cushion occlusal loads. PEEK-based restoration in implant prosthetics can compensate these occlusal forces, facilitating cushioning while chewing.

**Conclusion:**

This procedure provides excellent elasticity and resembles natural tooth structure. This clinical case suggests that PEEK restorations can be used in zirconia implants in dentistry.

## Background

In the field of implant dentistry, the most widely used implants over the past 40 years are those manufactured from titanium [1], which are still the most popular.

The recent demands for materials without metal alloys in dentistry, together with the increased sensitivity and allergies of some patients, have promoted the development of new materials.

An example of this is zirconia-based dental implants, known as zirconia or zirconium oxide implants. Its biocompatibility and its extraordinary mechanical properties make it suitable for numerous situations. Its main advantage lies in its elasticity which is greater than that of titanium and much greater than that of cortical bone. To avoid overload of the underlying bone from the direct transmission of biting impacts, several materials that can absorb part of this excess force have developed.

One of the prosthetic options lies in the combined use of PEEK restorations with composite coating on zirconia implants due to their physical and mechanical properties and their biocompatibility.

## Case presentation

A patient who is a 45-year-old woman and non-smoker has no medical record of interest. The patient complained of pain in the right second upper molar. She said that she felt intense pain while chewing. The pain was accentuated with occlusion and while chewing, making normal functioning impossible. The patient mentioned the absence of piece 16, which had been extracted 8 years previously.

**Fig. 1:**
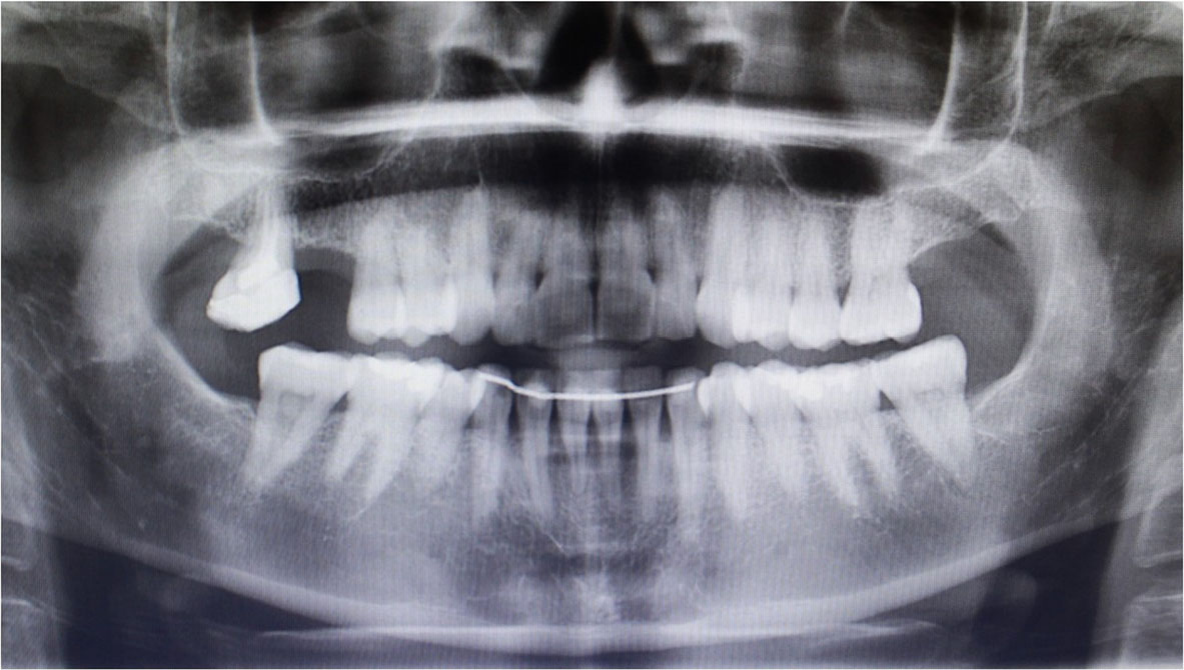
Diagnostic radiographic exploration previous to treatment

Clinical examination showed a longitudinal fracture in the palatal root of molar tooth 17, which was confirmed by a radiographic examination (Fig. 1), which was removed during this appointment. Due to the absence of both molars, the patient expressed a desire to replace them with implants. She also worried about having metal in her mouth and insisted on an alternative material to titanium implants, as well as her intention to replace her molars with metal-free restorations. After 4 months of healing, we proposed as a treatment 2 white SKY (Bredent®) zirconia implants, (4.5 × 10 mm and 4.5 × 8 mm), with PEEK restorations and composite coating. The patient was informed about the intention to publish the results and agreed that the data from this study were public. The patient accepts the treatment and signs informed consent. The CIROM clinical committee has approved the oral surgery for Zirconia and PEEK implantation to the patient.

### Initial exam

The edentulous crest showed an adequate amount of attached gingiva, thick enough to perform a flapless technique using a circular scalpel, [2] allowing the integrity of the peri-implant structures to be maintained, while diminishing post-operative pain [3].

### Surgical technique

**Fig. 2:**
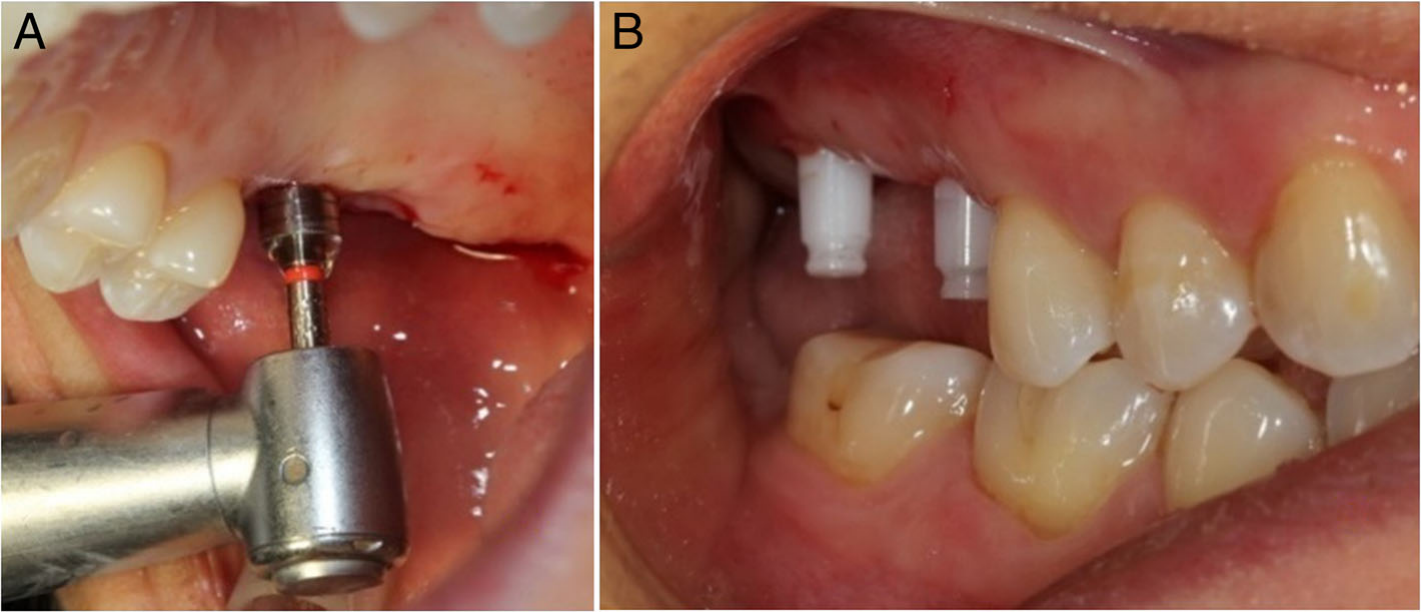
Flapless surgical technique, atraumatic surgical procedure for zirconium implants using the circular scalpel (**a**)–sharp, clean cut without bleeding (**b**)

For the flapless technique, we made two circular punch incisions in the gums, with a circular scalpel. The mucous plug was withdrawn with a periosteotome while maintaining the integrity of the gum around the incisions. We continued with the drilling indicated by the manufacturer, to insert two white SKY (Bredent®) zirconia implants of 10 mm length × 4.5 mm diameter in positions 17 and 16 (8 mm length × 4 mm diameter) (Fig. 2).

### Healing period

Fifteen days after surgery, the appearance of the soft tissue was excellent, with no signs of inflammation in the mucosa. The patient mentioned the absence of bleeding and pain during the post-operation period. At the same time, we made a clinical and radiological evaluation. Three months after surgery, the stumps of the implants were carved to improve their parallelism with a special diamond drill (Kit Bredent®). Finally, we took impressions for the final restoration with polyether (Impregum, 3 M ESPE) without using retraction threads.

**Fig. 3:**
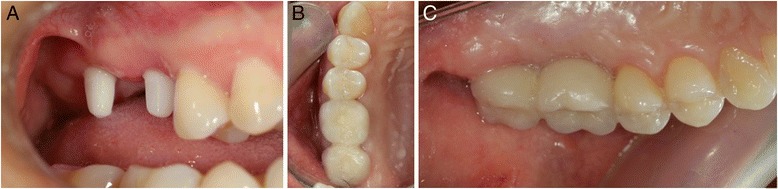
Final restaurations: The parallelism of the implants is achieved by carving the non-submerged part **a** occlusal view and **b** lingual view

**Fig. 4:**
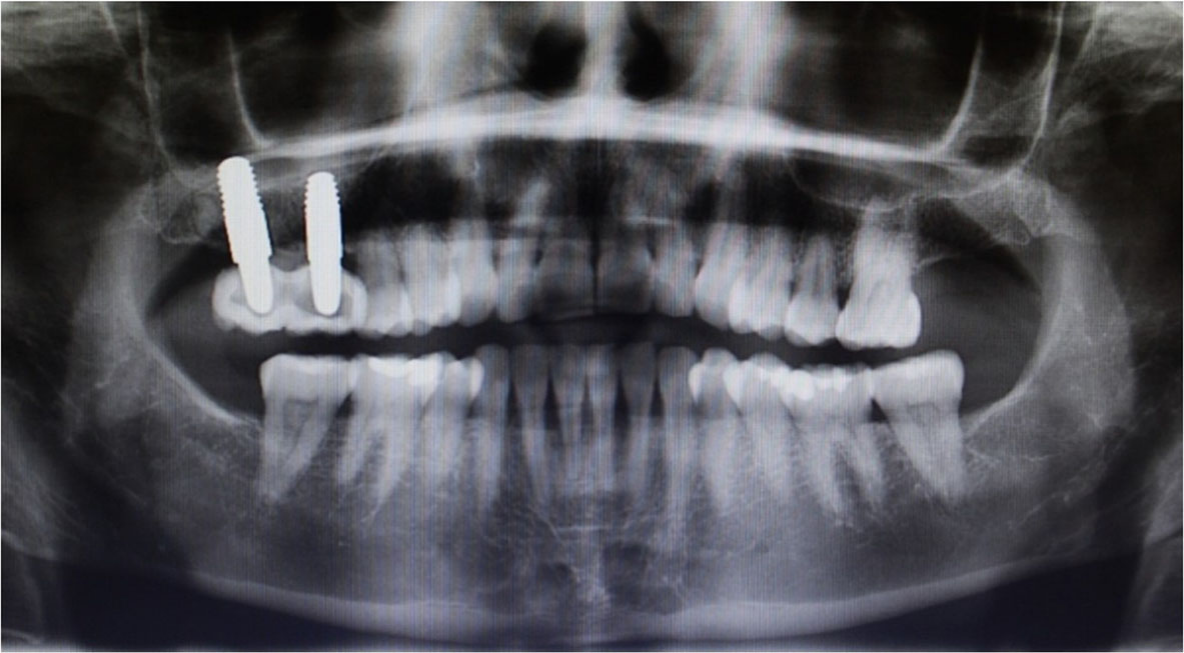
Follow-up after 1 year, no radiographic sign was appreciating and the osseointegration was satisfactory

The final restorations were produced using CAD/CAM System Juvora® for the PEEK structure with composite coating (Anaxdent®). For cementation ionomer glass cement reinforced with resin was used (GC FujiCEM, GC Europe N.V.) (Figs. 3 and 4).

### Tracking

**Fig. 5:**
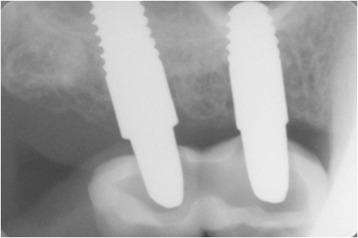
Periapical X ray after 1 year of follow-up, the bone was stable and no sign of peri-implantitis was shown

A clinical and radiographic review carried out a year after the initial surgery showed the complete success of the procedure according to Albrektsson’s criteria and the natural aspect of the soft tissue around the restorations (Fig. 5).

### Discussion

Intraoral conditions (saliva pH, acidic drinks, bacterial plaque, etc.) interact with metals, increasing corrosion, a phenomenon that also affects titanium implants [4, 5]. Amongst other reasons, this is whereby patients increasingly request the use of materials free of metallic alloys. In response to this growing demand, zirconia implants are considered an alternative, due to their low reactivity [6].

In recent years, several implant manufacturers have investigated the behaviour of zirconia implants on hard and soft tissues. The characteristics of their biocompatibility, together with good osseointegration, make them clear candidates for clinical use in dentistry [7, 8]. One of the advantages of these implants is the absence of cracks (gap) between pillar and implant since they are made in a single block. (Bredent®, Straumman®) [9, 10]. However, this feature implies the need to carve the pillars to achieve proper parallelisation.

Several studies have shown that zirconia implants present a similar healing pattern to titanium implants, both as regards the healing time and marginal bone stability [11–13]. However, there is a controversy over the long-term stability of the bone-implant interface, which depends on several factors such as surface, composition and design of the implant. Other important factors to consider are the implant-stump-crown connection, as well as the composition of the restorative material and the occlusal load transmitted by the antagonist tooth.

In terms of the load-cushioning capacity of the prosthetic elements, the use of PEEK as a prosthetic structure on implants has increased in recent years [14]. PEEK is a high-density thermoplastic polymer with a linear aromatic semi-crystalline structure that has exceptional physical and chemical properties as regards toughness, hardness and elasticity. Also, its low molecular weight, combined with the absence of metal, allows its use as excellent biocompatible prosthetic denture material.

PEEK has a modulus of elasticity (E-modulus 4 GPa) great overdenture implants compared to other conventional materials such as titanium (E-module 110 GPa) or zirconium dioxide (E-modulus 210 GPa).

In addition, the bending resistance of metal-ceramic restorations stands at around 400 to 600 Mpa. [15], in contrast to new composite coatings that have a Vickers hardness of approximately 400 MPa and a bending capacity of 314 MPa. Conversely, zirconia is three times harder (1200 HV) and its resistance to bending is 1400 Mpa [16].

As a whole, all of these features mean that the use of materials of high rigidity will result in direct transmission of chewing forces to the zirconia implant. This potential overload could cause bone reabsorption around the implants [17]. Some authors claim that this relation only exists in cases accompanied by a previous inflammatory process (of infectious origin), [18] where bone loss would be accelerated.

To avoid exceeding the adaptive limits of the bone and maintain the proper stimulation of mechanical stress that will keep the bone vital [19], PEEK components seem a viable alternative to obtaining a similar modulus to that of cortical bone. In this way, bone could be stimulated, favouring remodelling without overload [20]. It would concentrate the load by absorbing and distributing the same [21]. Its capacity of load absorption has led some authors to recommend its use in patients with severe bruxism [22].

Finite element analysis suggests that maximum contact pressure at the bone-titanium implant interface can be significantly reduced by using a PEEK crown rather than a ceramic crown [23].

In addition to PEEK, new coatings based on PMMA or composite materials (Anaxblent®Anaxdent®, Nexco®Ivoclar®, Solidex®Shofu®, Novo.lign®Bredent®, etc.) which incorporate ceramic fillings have been developed. Due to their molecular structure, these materials have excellent density and homogeneity [24]. The micro filling integrated into the polymer matrix increases abrasion resistance, at the same time as providing optimal elasticity which resembles the natural structure of a tooth. Although these restorations show good colour stability and a long-lasting shine, texture and brightness, they differ substantially from ceramic coatings which have excellent optical properties that enable them to achieve better long-term aesthetic results.

None of the authors have any competing interests in the manuscript. All authors have performed important contribution and have read and approved the final version to be published.

## Conclusions

Zirconia implants with PEEK restorations can be considered a good alternative for replacing natural teeth. Their biocompatibility and biostability make them a promising material for those patients who suffer from allergies and sensitivity to metal alloys.

PEEK restorations are a valid and alternative recommendation when using zirconia implants because of their cushioning effect and elastic modulus, which absorb occlusal forces and wear like a natural tooth, which could optimise and preserve osseointegration with time.

Within the limitations of this study, we recommend the combined use of zirconia implants, PEEK structures and PMMA coatings in patients with intolerance to or rejection of metal alloys.
